# Supplementary material for: Loss of a major toxin gene cluster defines a metabolic schism and host-specific virulence in Botrytis pseudocinerea
Source: PLoS One. 2026 Mar 24;21(3):e0339017. doi: 10.1371/journal.pone.0339017 (PMC13012479; doi:10.1371/journal.pone.0339017)
Supplement: Attachment — Submitted filename: Response to reviewer 2.docx [file pone.0339017.s003.docx]

**Dear Reviewer 2,**

Thank you for your encouraging assessment of our manuscript and for recognizing the value of our data regarding secondary metabolite production and host-specific virulence. We appreciate your highly specific technical and scientific critiques, which have significantly improved both the visual presentation and the genomic depth of our study. We have carefully addressed each of your points in the revised manuscript as detailed below:

**1. Comment regarding the low resolution of Figures 1-3 in the PDF output:** *Response:* We apologize for the difficulty in viewing the figures. The degradation of image quality is a known artifact of the automated manuscript compilation software utilized by the editorial system, which often applies aggressive lossy compression to generate the reviewer PDF. To completely circumvent this issue, we have fundamentally altered the format of all figure files for the resubmission. All figures have been re-rendered from their original high-resolution source files and processed through PLOS ONE’s Preflight Analysis and Conversion Engine (PACE). We have uploaded them as standalone, high-resolution (minimum 300 DPI) TIFF files utilizing lossless LZW compression. This guarantees that the visual evidence will be perfectly preserved and clearly viewable in the final publication.

**2. Comment regarding the clarity of Fig. 3A (gerbera petal assay):** *Response:* We completely agree that the original photographic panel for Figure 3A lacked sufficient depth of field and contrast, making it difficult to clearly see the petal margins. Following your direct recommendation, we have entirely replaced Figure 3A. We conducted a new set of gerbera petal infection assays and photographed the infected petals at 72 hours post-inoculation using high-resolution macro-photography. Crucially, the new imaging utilized oblique, directional lighting to highlight the textural differences between healthy epidermal cells and the collapsed, macerated tissue of the necrotic lesion. The margins of the infection are now starkly delineated, providing unambiguous visual confirmation that the virulence of both strains is equivalent on this baseline host.

**3. Comment requesting clarification on the downstream enzymes of the botrydial pathway and the suggestion of PCR analysis:** *Response:* This is a highly perceptive question that cuts to the core biochemical mechanics of the metabolic schism we observed. You correctly noted that the accumulation of mevalonolactone proves a blockage, and rightly asked which specific downstream genes are missing or mutated.

While you suggested a targeted PCR analysis, we decided to provide a far more robust and comprehensive answer by bypassing PCR (which can be prone to primer-binding artifacts in divergent cryptic species) and instead performing an exhaustive *in silico* comparative genomic analysis using the newly available *B. pseudocinerea* reference genome (strain Bp36).

Our analysis yielded definitive answers, which have now been fully integrated into the revised Discussion section:

- We confirmed the presence of highly conserved, intact orthologs for the upstream mevalonate pathway, including the essential mevalonate kinase gene (*ERG12*). This explains the organism's capacity to synthesize the massive quantities of the mevalonate precursor.
- However, our search for the committing enzyme, the *BcBOT2* sesquiterpene cyclase, revealed that this specific gene is not merely mutated, but completely physically absent from the genomic locus.

This specific genetic deletion prevents the cyclization of farnesyl diphosphate (FPP) into the botryane skeleton, creating a severe metabolic bottleneck that causes the upstream mevalonate to accumulate and spontaneously lactonize into the mevalonolactone we isolated. By utilizing this *in silico* whole-genome mining, we hope to have provided an exhaustive and definitive resolution to your excellent inquiry.

Thank you again for your time, your constructive review, and for helping us elevate the quality of this manuscript.

Sincerely,

The Authors
